# Book Reviews

**Published:** 1808-02

**Authors:** 


( To be continued. )

---

### CRITICAL ANALYSIS
### OF THE
### RECENT PUBLICATIONS
### ON THE
### DIFFERENT BRANCHES OF PHYSIC, SURGERY, AND MEDICAL PHILOSOPHY.

---

*On Fever, as connected with Inflammation: An Exercise.* ———, CLIFTON. 1807.

NEVER was a more appropriate title affixed to a book, and the motto from Hippocrates is not less pointed. Would Dr. Beddoes have ushered all his unfinished productions to the world in the same manner, he might have been excused for the crudity of some. Perhaps the titles of the others might have been improved; but the present may fairly claim the name of an Exercise. As such we shall consider it, and begin with freely acknowledging our obligations to the author for the diligence with which he has accomplished his task.

After some lively remarks on the manner in which medical Inquirers have directed their researches, and their general dissatisfaction from the want of well recorded facts to guide them, or the obscurity in which those facts are involved, the subject is introduced by a short chapter on the connection of fever with inflammation. In this we meet with a very general view of what is technically called symptomatic fever, and at the threshold discover a hastiness to proceed, which is only excusable, by reflecting that the whole is an Exercise. On this account, we shall only copy the passage, and leave the author to amend it hereafter. "Thus we have *quickened pulse* with shiverings and flushings, *when the*

*the stomach is inflamed from poison;* the *kidney from a stone;* a *bowel from strangulation*, or the gums from a carious tooth."

The succeeding chapter is as important as it is satisfactory, in showing the "connection of inflammation with fever," of whatever description, and almost in whatever stage. After these general remarks, the author proceeds to consider the "dependance of fever (idiopathic fever as it is called) on inflammation of one particular organ. To show that this affection may be seated in the head, an account of an epidemic at Aumale is extracted, as given by Granvilliers, in which the whole disease is referred to inflammation in the brain, or congestion in that organ. Another history follows, of an epidemic at Wetzlar, as related by Winlestadt, in which all the various symptoms of typhus, the nervous, and the bilious fever were traced to increased action of the vessels about the brain. In both, the only remedy was repeated, and copious bleedings.

"The first author did not extend his ideas beyond the epidemic before him; he does not, we see, even attempt a rationale of the later stages of the disorder, as depending on the first. The second, though he points out the way in which the secondary affections arise out of the original inflammation of the brain, extends his principle no further than to say, "that unquestionably, a putrid fever, as it was called, prevalent at Wetzlar in 1770, and the years following, was this same fatal complaint; at which time," he adds, "it is too certain, that many a patient paid with his life for the error of his physician."

"When once in such a train, the theory could not fail to be generalized. The wonder is, that its influence on books of general practice and nosology, should have been so tardy. Several years ago I had learned, by oral information, that it was taught by a Professor of Medicine in the university of Tubingen, and at length, with some difficulty, I procured for perusal two academical tracts, entitled, one, *Diss. sistens expositionem nosologicam typhi*, *Tubingæ*, 1800; the other, *Diss. sistens therapiam generaliorem typhi*, ibid. 1801. Both were defended under the presidency of Dr. Ploucquet, and derived, no doubt, from his instructions; the former being avowedly corrected by him, in order, as he says, "that it might agree more perfectly "with his nosological principles."

"Compleatly to exhibit the progress of this doctrine, I should state that Dr. Rush describes the brain as principally affected by congestion in yellow fever, and that even in 1793, he took one of his strongest indications from this idea. And did I not foresee through what a labyrinth of quotations I am destined to lead my reader, I should likewise introduce passages from Bonetus, Lieutaud, Stoll, Tode, and Dr. Pew. But their sentiments have been stated either by Burserius or later writers; nor do those sentiments stand so instructively connected with decisive practice. I shall, therefore, pass on to a very extensive "*Enquiry into the seat and nature of fever,*" which, preceded by an inaugural dissertation, was published a few weeks ago by H. Clutterbuck, M. D. and, in reality, ality, contains the same opinion, supported by the same proofs as Dr. Ploucquet employs."

The author proceeds to an examination of each; and it must be admitted, that the coincidence is striking. We have not a doubt, that Dr. Clutterbuck will feel himself obliged by the production of this confirmation of his ingenious hypothesis, especially as it is remarked with much candour, that there is no reason to suspect any plagiarism. It may be considered by some, and perhaps our author would lead us to such a determination, that a theory broached, and afterwards neglected, may presumptively be considered as ill founded. But he should recollect, that he was among those who found that pneumatic chemistry was almost discovered by Mayow, though neglected by most of his successors, and certainly unknown to Priestley, Cavendish, or Bergman.

The opinions of Drs. Ploucquet and Clutterbuck are afterwards formed into a parallel, and the author concludes with remarking, that the former are conveyed in much less space than the latter. It would have been candid to acknowledge, that the English physician has taken more pains to remove all the objections that may be started against his theory. Some general remarks follow on the importance of morbid anatomy, interspersed with not a few on the uncertainties attending it. Enough however, it is said, may be learned from such records to justify our inquiries into the labours of others; and it would be great injustice to Dr. Beddoes not to acknowledge the obligations we owe him for the evidence he has accumulated of recorded dissections of febrile subjects. For this purpose we have an analysis of what has been given of an epidemic in Geneva, in Leipsic, in a part of Normandy, and at Leghorn. In all these we meet with diseases in the abdominal viscera as well as in the head. Returning to America, we are told, that Drs. St. Ffirth and Mitchell always found the stomach diseased. But the former, as well as Dr. Rush, fonnd the blood vessels of the brain turgid, and occasionally adhesions between the dura and pia mater. We must also remark, that this appearance, so constant in the stomach, may, without any violation of language, be imputed to that solution of the stomach which Hunter proved the effect of digestion after death. "Erosions of the villous coat frequent, in a number of cases detached pieces as large as a dollar floating in the black vomit: blood vessels very much distended." The latter, it is true, may be considered the true sign of inflammation; but it must be remembered, that Mr. Hunter particularly remarks, that where the villous coat is digested the blood vessels are exposed to view.

We have next a collection of morbid anatomy in brutes who died under epizootic maladies. For this, while we thank the industry of Dr. Beddoes, and of those diligent observers from whom he produces them, we cannot help blushing when we reflect how little has been done at home in this as well as the former most important research. In both the selections are from foreign writers, chiefly chiefly Journals. Above all, let it be known, that the College of Health at Pavia thought it right a report should be drawn up of an epidemic among cats, with the result of such dissections as were well authenticated.

From the various testimonies deduced under epidemics and epizootics, the author does not scruple to assert, that in idiopathic fever the stomach and contiguous parts have been found more constantly and deeply affected with inflammation than the brain and its membranes. We must, however, again remind him of the solution of the stomach after death, particularly as he offers a case, in our opinion, much to the purpose, in answer to Dr. Home's case, on which Dr. Clutterbuck dwells with so much propriety.

"There stand, says Dr. B. on record single cases, watched with more than common care during their progress, and more nicely investigated after death. These, to say the least, appear not unfavourable to my conclusion. To the observation of Dr. Home upon himself, I shall oppose another, in which the parties concerned must inspire the highest confidence, as to what regards both pathology and anatomy. An observation of this character is worth a host such as are less particularly made, and by persons of inferior ability.

"The case I have in view is that of Dr. I. F. G. Goldhagen, as related by the present Professor Reil, in a small tract published at Halle in Saxony, in 1788. During the prevalence in that town of a fever, styled nervous, Dr. Goldhagen was seized, in consequence, as is believed, of contagion, received from some patient. Lassitude, rigour, biting heat, anxiety about the præcordia, prostration of strength, severe shooting pains in the head, stupor, delirium, tension without pain of the epigastric region, quickness of pulse, petechiae, were the chief among the symptoms that succeeded each other in 13 days. The patient treated himself with purgatives (so as to produce an American effect) and with emetics at first. He afterwards had vitriolic acid, valerian, serpentaria, camphor, musk, blisters, sinapisms, stimulant liniments, fomentations. The earliest lassitude and general state of uncomfortable feeling occurred after a night of unrefreshing sleep, on the morning of December 29th, 1787. It succeeded to an evening of unusually high spirits. During the night ensuing, he felt the febrile chill. In the morning he procured many stools. In the evening occurred several irregular feverish accessions, beginning with chills, which were succeeded by more lassitude than heat. The pulse soft, full, and of the natural frequency. On the 10th of January, 1788, he died.

"The body was next day opened by Dr. Meckel. The exterior exhibited strong marks of putrefaction. On opening the abdomen the small intestines burst out with force. They were so distended with wind as to have pushed the large intestines (also distended) back into the posterior parts of the abdomen and up to the diaphragm. The coats were transparent; thinner perhaps than in in the most reduced consumptive subjects. In two or three places were found such contractions as lessened the area at least one half. In the descending colon, above its curvature in the left side, was a place that would scarcely admit the little finger. Hardly any vessels visible on the intestines, yet here and there on the small intestines and left curvature of the colon were large bluish spots. In cutting them up, the whole cavity contained two or three consistent, though soft pieces of fæces, of the size of a wallnut. About a pint of brown offensive liquid flowed out. The large intestines were very wide and tender, on the inner suface appeared little reddish spots, to which fæces adhered so firmly that they could scarcely be abraded by the scalpel.

" It was only when the bowels were removed that the stomach and liver came into sight. The liver was grey, tender, and small; the gall-bladder not distended by bile; the stomach empty, and so collapsed that the spleen was perceived projecting at its bottom. On its anterior surface was a fissure two inches long, with a tender, thin, white margin, looking and feeling as if dissolved by putrefaction. On cutting the stomach up, the coats near the fissure were soft and extremely thin; and at the fissure quite destroyed. At some distance, the vessels of the villous and nervous coats were turgid with dissolved, dark blood, which, in places, had become effused into the cellular substance.

" The brain was very firm, the vessels moderately full of blood. Between the membranes, and in the ventricles, there was a little colourless liquid.

" In the broad, well-formed chest, the lungs lay without adherences, and expanded with air. In the cavity of the thorax, especially in the left, there was a considerable quantity of dark-red serum: and a strong smell of musk arose from this cavity. On turning back the heart, the œsophagus, from the diaphragm to beyond the *atrium venarum pulm.* was so sphacelated in its whole circumference, that scarce did there exist the smallest coherence between the fibres.

" Dr. Reil, the assiduous and acute friend of the deceased, thinks the contractions in the bowels were of long standing, and the effect of posture.

" Goldhagen, he says, rose in summer at four, and in winter at five, and studied till near midnight. He did not interrupt his labours for supper. When reading, he sat upright. But in writing, and he wrote a great deal, he stooped; his head hanging forward, and his abdomen compressed. He did neither standing. The strictures then arose from the bowels being frequently and long compressed.

" Be this as it may, it appears evident where inflammation had and had not been active. I am not sure that the expression *very firm*, as applied to the consistence of the brain, is meant by the celebrated anatomist to convey the idea of disease. If it were, I can produce reasons for thinking that it might have had nothing to to do with the fever. The pamphlet fortunately contains a history of the patient. He was a man of anxious mind, of indefatigable application, and addicted to profound meditation."

Dr. Beddoes proceeds to show, and we are not disposed to dispute the point, that the former habits of the patient *might* have produced this firmness of the brain. Perhaps he may impute to the same cause the small quantity of colourless fluid found between the membranes and in the ventricles. But admitting all this uncertainty, where are the proofs of mortal disease in any other organ? During the fever, *severe* shooting pains in the head were felt, with stupor and delirium; biting heat, anxiety about the præcordia, tension without pain of the epigastric region.

The body, though opened the day after death, exhibited signs of putrefaction. Such a state was favourable, according to a late discovery, to the digestion of the stomach. Though all the intestines were distended with wind, the stomach was collapsed and empty. To what are we to impute "a fissure two inches long, with a thin tender margin, looking and feeling as if dissolved by putrefaction." Some apology may be allowed for those who were ignorant of Mr. Hunter's discovery, of which Dr. Beddoes speaks in another place * with so much respect.

These might, at least, hint a modest conjecture concerning *solution by putrefaction* in a few hours. But in what follows, no such conjecture can be offered. "On cutting the stomach up, the coats near the fissure were *soft*, and *extremely thin*, and at the fissure quite *destroyed*. At some distance, the vessels of the villous and nervous coats were turgid with dissolved dark blood, which in places had become diffused into the cellular membrane." That is, to use the language of Mr. Hunter, absolute universal death having taken place, the blood did not coagulate, and the stomach was digested: in some parts through its whole substance, in others in such a degree as only to expose the blood vessels; in others the vessels were digested, and the blood remaining fluid, or as it is here termed, *dissolved*, escaped into the surrounding parts. In our opinion, therefore, the presumption of inflammation is stronger in the brain than in the stomach.

Hitherto, we have endeavoured to follow our author step by step, but to continue such an attempt would be to transcribe the book. We are kept in hard *exercise*, even to examine what *he* has accomplished with seeming ease. We shall therefore haste as fast as is admissible

---

* "Changes for the greater and the less happen according to the season and the nature of the case, within a few hours after death, in a day, or not till later. These, if regarded by themselves, would sometimes denote too much, and sometimes too little. The solution of the stomach is an instance on one part; on the other, the observation of Bichat, (amply confirmed by others, and nearly the most interesting in pathological anatomy, since the discovery of Mr. Hunter relative to the stomach) that *inflamed serous membranes soon lose their redness*." See page 44.

admissible to the most important part of every medical work, viz. the practical inferences.

After a few judicious remarks on the effect of inflammation on the whole system, according to the nature or function of the part inflamed, the internal appearance in other diseases is contrasted with what has been described in fevers; and the progress of inflammation is traced, whether continuous or diverted by metastasis, to different, and sometimes distant parts.—The various periods of fever, at which inflammation may commence, are next pointed out with much ingenuity, and with the same weight of authority as was produced in support of the former suggestions. "Some analogical considerations" follow, showing the probability of inflammation, in other diseases in which it has not at present been sufficiently suspected. Hydrophobia occupies the most of this division, and the importance of the subject more than justifies the length of the author's remarks and inductions.—A section on the "evidence from the sensorial functions" follows, principally to show that mental derangements are not necessarily the effect of inflammation, or any visible alteration in the brain. After some useful remarks on Hydrocephalus Internus, the author takes a transient view of the "fluctuation of opinions and practice in fever."

This section contains many valuable remarks on the invariable apprehension which the physicians for the last half century have expressed concerning *debility* in fevers; and points out the great importance of blood-letting, exemplified in the success of many of the continental physicians, as well as of Rush and others in America; of Jackson, in England, and many other parts of the world.

"Dr. Jackson, (says our author) who from his experience in so many climates, may be styled the Ulysses of medicine, had pursued in certain varietes of fever, and those the most malignant of all, that practice of profuse blood-letting, by which Galen extorted from the astonished spectators, the gratifying exclamation, εσφαξας, ανθρωπε, τον πυρετον. *Oh man, thou hast cut the throat of the fever!*—and which, *seasonably employed*, has been attended with the same instantaneous success in the hands of the ablest physicians of every age. In an instance which he particularizes (*Med. department of the army*, p. 145,) by at once drawing 56 ounces of blood, he relieved the patient, as he himself expressed it, "*from chains and horrors;*" so that by the addition of a blister and emetic tartar with opium, the danger, observed also by another physician, was past in four hours. He had sometimes used blood-letting, as a preliminary to warm and cold bathing. Of the "very important differences between Dr. Jackson and himself," Dr. Currie speaks in a tone as nearly approaching to censure as the unaffected benevolence of his nature would allow. He is not "surprized at the imperfect success of the cold affusion in the hands of Dr. Jackson," (ii. 197) who requires a high state of excitement or of sensibility on the surface in its application.—For inferiority of of success, if the fact were allowed, the far greater virulence of disease in the patients which the army physician has to treat whether at home or abroad, will, I think, go no inconsiderable way towards accounting. But Dr. Jackson, (p. 130—133) makes an attempt, by no means unsuccessful, to shew that the mortality in his hands was actually less than of the same military hospitals under other treatment, and less than in London; less even than in the workhouse at Liverpool, as reported in Dr. Currie's own first publication.—And in extensive malignant epidemics, blood-letting under a proper application seems to have been just as successful as cold affusion in the most favourable examples: a fact which shews the relative excellence of both methods.

" Dr. Jackson observes (l. c. 240) " that where there are marks of bloated stagnation and inability—torpor, sluggish, languid and oppressed circulation, countenance dull, difficulty in expanding the chest, bleeding will restore the susceptibility of impression," the doctrine seems not only intelligible but salutary. For in the varietes of common fever of this country these affections exist not unfrequently in a degree, and are removed by free bleeding about the head, and cold applications to it."

Let the reader contrast this, with the cautious manner in which the cold affusion is afterwards spoken of.

" But after weighing, (says our author) the conjoined testimony of description, dissection, and experience, so far as the latter goes, I shall be most agreeably surprised: if the *affusion of cold water, such as it is taught by Dr. Wright and his able commentator, shall ever establish itself as the common remedy for that fever, which has lately ravaged America and the warmer regions of Europe.* It may do very early and in the slighter cases. In some of those fevers of our own climate, which put the healing art to the test, it will scarcely be effectual, unless perhaps employed before any one can pretend certainly to say what the case is. Now a bad case on its approach may be regarded as equivalent to a slighter fever fully formed. In the *medical reports* themselves, one infrequent variety is pointed out as an exception to the cold affusion.—A young gentleman (whom Dr. Currie would have lamented from his long intimacy with members of his family,) at a time and in a condition of life, in which typhus is commonly least dangerous, died last spring, though his method was employed under the direction of two very able physicians. Among persons, ill of typhus, whose cases have been stated to me, or whom I have seen during the present year, he was the only one who perished, (as I suspect from organic disease) and the only one so treated. For in the rest, local inflammation, diarrhœa, suppression of bile, pregnancy, or the late period of the complaint, excluded this capital remedy. He appears to have received the complaint by contagion. Dr. Clutterbuck states from his own observations in 1803, that pulmonic and rheumatic inflammation had been so frequently excited by the cold affusion in the Glasgow Infirmary as to bring the remedy into some degree gree of disrepute; though the situation of the patient was never rendered materially worse by this accession of disease. Dr. Jos. Frank, who conversed much with Dr. Currie at Liverpool, and saw the practice successfnlly employed in the house of recovery in London, introduced washing with cold water into his department of the Vienna Hospital, (for he was apprehensive lest the affusion should excite alarm.) He gives an account of various successful cases, in one of which, however, he doubted if the cold did not produce pain of the chest and cough—adding, that in a similar fever he had twice seen iced water applied to the head, produce a fatal pneumonia, (*Reise* II. 288—96.) Since the departure of Dr. Frank for Wilna, cold applications appear to have been discontinued. His pupils represented the practice as little successful, (*Rec. Period.* xxvii. 412;) probably on account of some occasional inconveniences.—The Germans seem peculiarly subject to certain rheumatic affections. I am told their soldiers proved so in England. This would deserve consideration in typhus.*"

After this, we cannot but be surprised to find a proposal for a "remuneration to Dr. Currie; not forgetting Dr. Wright," but forgetting Dr. Jackson! who has taught us the mode of conducting these affusions, and the previous cautions which Dr. Beddoes seems to admit, are indispensible. When Dr. B. says, "the want of rules, such as we owe to Dr. Currie, is deeply to be lamented; he cannot surely mean to imply, that the thermometer is to be a sufficient guide in so important a remedy. If heat is among the marks of inflammation, and it is necessary, under inflammation, to premise bleeding to the cold affusion, how dangerous a test for its immediate application must the thermometer alone prove.

Though we have marked these inconsistences in the work before us,

---

"* Accounts of the Edinburgh clinical practice, in that most important case of fever-patients affected with pneumonia, do not quite agree. Frequent trials, according to Dr. Reeve, afforded most satisfactory results on this subject. "Not one of the patients who had symptoms of catarrh or inflammation of the lungs, suffered the least inconvenience from the cold or tepid affusion." Currie II. 96. Of the same patients with some more, Dr. Keith, clerk to Dr. James Home, says, that sometimes cold affusion could not be freely employed on account of vehement catarrhal symptoms (*de aquæ usu ext.* 1804, *p.* 38;) in paucis exemplis affusio frigida nullam vel in pulsum vel in calorem vim habebat. In adhuc paucioribus *hos augebat* et in his exemplis solummodo, *ubi symptomata catarrhalia admodum valebant.*" ib. p. 39. This last account appears to be confirmed in Dr. Lampert's thesis, written at the same time, p. 21. Dr. Reeve himself tells us that blistering and bleeding were practiced; qu. if before the cold dashing? and that where the catarrhal symptoms were strong, tepid affusion was preferred. It appears therefore that the cold disagreed with some, and that its disagreement with others was feared. I have never dared to recur to it in such cases without leeching at least and blistering. All this tends to confirm Dr. Jackson's practice, in which too inflammation probably ran still higher."

us, we are ready to admit its value. Indeed, we have never seen from the able pen of Dr. Beddoes, a work which contains a collection of so much useful matter, so well digested;—a work, which must impose upon the medical student, the necessity of thinking; which will "teach him, issuing warm from the lecture room, that he ought not to reject, on the strength of what he hears there, a contrary practice, as out of nature." When, however, we are in pursuit of such noble game, we must not content ourselves with ordinary toils, we must pursue somewhat closer in proportion as we respect the object in view. To drop the metaphor, we could wish Dr. Beddoes to revise his whole chapter on temperature. We have read it over more than once, and whenever we fancied we had discovered a thought, have hitherto found ourselves disappointed. We cannot say that the whole is unintelligible; but are obliged to confess, that the greater part of what is given as original matter, excepting the historical passages, requires further explanation for our limited capacities. Again, we could wish an extension of candour to a few more of our *living countrymen*, than Drs. Wright and Jenner. We must allow, that foreigners and defunct Englishmen, are well enough treated, and that Dr. Clutterbuck is, in some places, called ingenious, though he is said not to be original. Dr. Beddoes, indeed, does not propose *his* work as original, but rather as a collection of what has been done; but even in his connecting passages and inferences, there is less of originality than he may be aware of. We could show several good passages which might be called paraphrased from Dr. Jackson. Perhaps they were intended to be considered as such, though it might be thought unnecessary to repeat his name more frequently than it occurs. There is also another writer, the perusal of whose work, suggested our remarks on the digestion of the stomach, whose name does not occur in Dr. Beddoes pages; yet whose opinions, and almost whose words, are sometimes very similar. Dr. Adams, in accounting for the introduction of the warm stimulating plan in fevers, has the following passage. "At length, the loss of some individuals in a noble family, under scarlatina, alarmed the public; and ulcerated sore throat became synonimous with putrid sore throat. To this, by an easy transition, was added putrid fever; and TYPHUS was re-echoed from the professor's chair to the nursery."

Dr. Beddoes says, "The tract of Dr. Fothergill on the putrid sore throat had a vast influence in determining the public mind in favour of the stimulating treatment; and the Doctors of the Scotch school, whether professors or private teachers, whether friendly or hostile to one another, whether they founded spasm upon debility, or freed their doctrine from spasm as a vile encumberance, co-operated most strenuously in completing the work, which had so far been prepared for their hands. Putting *debility* in the place of putrescence, they rendered it almost the universal watchword of medicine, and annihilated for a time great part of the benefits of experience."

Dr. Adams, after urging the necessity of bleeding to prevent in-

 flammation flammation in fevers, adds: "All fevers, if violent, and attacking persons in high health, sometimes produce mortification in the intestines, and if long protracted, mortification will occur in superficial parts."

Dr. Beddoes. "In fact, fever will *naturally* always end more putrid, as it has begun more inflammatory, and frequently will be more nervous at the same time."

Our only intention in the production of these passages, is to show, that if Dr. Clutterbuck has thought like a foreign physician, Dr. Beddoes has also thought like an English physician. The thoughts are too unimportant to be considered otherwise than as the natural effusion of each writer; but the coincidence is worth remarking.

With these few hints, we take our leave of this very useful work, heartily rejoicing, that whilst whatever objections Dr. Beddoes may offer to Dr. Clutterbuck's theory, and, however Dr. C. may be disposed to receive those objections, both join with many other respectable writers, in endeavouring to rescue our patients from that dreadful routine of bark and wine, which has too long engrossed the practice in fevers.

---

*Colloquia, Anatomica, Physiologica, atque Chemica, Questionibus, et responsis in usum ingenuæ juventutis accommodata.* Auctore ARCHIBALDO ROBERTSON, M. D. Edinburghi e prelo academico. 1808.

IF this book is intended for the use of the ingenuous youth of that academy, we cannot speak so well of the ingenuousness of the author.

The preface might have explained the object of the work, in such a manner, as to meet this objection. Is it to be supposed, that young men will learn latin, in order to read a book which contains much less than they will hear from their teachers. Rather will they not be facilitated in learning from this book, just as much, and no more latin, than will be necessary to pass their examination *pro summis in medicina honoribus* RITE ET LEGITIME *consequendis*.

---

*A Letter on the Practice of Midwifery, addressed to ———, occasioned by, and including an Account of a late unfortunate Case, with some Observations and Reflections on the Subject.* By JOHN BOYS, Physician, Man-midwife to the Westminster General Dispensary, and Teacher of Midwifery in London.

THE unfortunate case above alluded to, is too well known to require any commentary; and the no less unfortunate practitioner has paid the penalty in being tried capitally for an ignorance certainly inexcusable, but not deemed criminal.

The principal object of the present publication is, to show the necessity of some professional examination of those who undertake this important branch of practice. This cannot be doubted, and we hope the event will induce the College of Physicians or Surgeons to add this to their other examinations.

### *Edinburgh Medical and Surgical Journal, for January,* 1808. *No. XIII.*

ARTICLE 1.—*Report from the General Hospital, near Nottingham.* By JAMES CLARKE, M.D. One of the Physicians to the Charity.

IN this report are two cases of Hydrocephalus; one in a married woman, 37 years of age, the other in a boy of 11. Some practical remarks follow on gastrodynia, but contain nothing very new.

ART. 2.—*Some Observations on the Diagnosis between Fever and Phrenitis, and on the Nature and Treatment of those Diseases.* In a Letter to ANDREW DUNCAN, Jun. from Dr. J. P. WILSON.

It is not to be wondered if Dr. Wilson is dissatisfied with Dr. Clutterbuck's opinion of the cause of fever, since, by the report of fevers in Worcester, *none* of them bear the lancet. This may appear strange to those, whose practice is confined to any particular district, or even to the metropolis. However, we have many reasons, besides his own accuracy and practical knowledge, for believing that Dr. Wilson is right. In those manufactures which are situated in old cities, and do not fluctuate with seasons or foreign demands, the work people remain inhabitants of close ill-built and narrow streets or passages. Their wages are entirely at the mercy of their employers, who rarely have those sudden demands, which oblige them to give additional encouragement for labour. Hence the poverty of those engaged in such stationary employments becomes progressive, as the difficulty of living increases, and the air of their habitations becomes more deteriorated; as the extremities of the city increase, without the destruction of the confined parts, as has gradually taken in the metropolis. For this reason, we are ready to believe that the glovers of Worcester, and probably the pin-makers of Gloucester, are principally affected with the diseases of poverty; and that the proper physic for them, is what Rousseau called the physic for the poor in general, namely, wine. We are, however, much surprised, to find the author of this paper remarking, that he has never found an instance of fever without accelerated pulse.

In the arguments produced to distinguish fever from phrenitis, Dr. Wilson has no difficulty in showing that a state of phrenitis, in which the brain or its membranes are highly inflamed, is early distinguished from the fevers of Worcester. But we should recollect that there are inflammations, which will not be relieved without tonic remedies: of these kinds are erysipelas in delicate subjects, and even some phlegmonous appearances, which neither resolve themselves, or come to suppuration, till the constitution is assisted, or spontaneously recovers itself.

We mean not by this to engage in a controversy, which is already in able hands; but to show that the question is somewhat more complicated, than Dr. Wilson seems aware of.

At the close of this paper, the author takes an opportunity of stating his own opinion of the proximate cause of fever, which, he says, differs from Dr. Cullen's; inasmuch as the latter rests solely on the *vis medicatrix naturæ*, whereas Dr. Wilson explains the same effects by the known laws of the animal economy.

"Dr. Cullen," it is added, "attributes nothing to the excrementitious matter retained, in consequence of the debility of the excreting organs; on which, if my opinion be just, a great part of the phenomena of fever depends."

Let us see how this difference is maintained. Dr. Cullen speaks of spasm on the extreme vessels; Dr. Wilson of debility, by which the extreme vessels are unable to rid themselves of excrementitious matter. Dr. Cullen supposes that the spasm induces an attempt in the constitution, at overcoming that cause which impedes its actions. Dr. Wilson, that the excrementitious matter thus retained, excites to preternatural action.

"The heart and larger vessels, which restore the action of the extreme vessels, in the same way as the increased action of the larger vessels of an inflamed part restores the action of its capillaries; that is, by increasing the quantity of the natural stimulus, viz. of the fluids impelled into them by the *vis a tergo*. The retained excrementitious matter is thus thrown off, and the preternatural action of the heart and larger vessels subsides. But if the debilitating power has been considerable, on the removal of the preternatural stimulus (the retained excrementitious matter), the debility of the vital system recurs, and the same phenomena are renewed. Thus, when the fatigue of the animal organs has been excessive, a series of febrile paroxysms is the consequence. When we see other debilitating causes affecting the vital organs, and producing the same train of symptoms, is it not a fair inference, that they operate in the same way."

Now it appears to us that *spasm*, or *retained extrementitious matter*, taken as a cause of fever, are equally without proof; and if Dr. Cullen calls in the *vis medicatrix naturæ*, we know not how Dr. Wilson can do without it, when he admits that preternatural action is excited under debility. All this, we admit, may arise from dulness on our part. To this imputation we are ready to submit, as long as we are not accused of misrepresentation, or a want of diligence, in unravelling our author's meaning.

Art. 3.—*Case of Malconformation in the Genitals, with an Engraving.* By Mr. John Smith Soden, Member of the Royal College of Surgeons, London.

This is one of the cases nearest to the hermaphrodite, that has come within our reading.

Art.

Art. 4.—*History of a Case of Diabetes Mellitus, successfully treated by Animal Diet and the Use of Cinchona; with Remarks.* By George Alley.

This is an useful practical case; and though not sufficient to establish, with any certainty, the permanent success of such a mode of treating diabetes, serves to confirm the propriety of the attempt. The concluding remarks are judicious. We shall extract the following, as offering a hint which may be improved.

"I shall conclude," says Dr. Alley, "this communication, by adverting to the place which this disease should have in systems of nosology. Dr. Cullen has placed it under the order of *spasmi.* This improper arrangement has been wisely accounted for by Dr. Willan, when he says, "most of the plans of nosology are exceptionable, as being founded on hypothetical principles, rather than a strict analogy between the diseases put in the same order." If we attentively consider the circumstances attendant on diabetes, the emaciation, debility, œdematous swellings of the legs and feet; and, in some cases (particularly towards the close of the disease), the quick pulse, and hectic flush, we shall be inclined to take the disease from the order in which Cullen has placed it, and rank it under the class of *cachexiæ*, and amongst the *marcores.* The *tabes sudatoria* resembles much, in its nature, diabetes; the former is attended with a diseased state of the primæ viæ, and cutaneous vessels; the latter with a diseased state of the primæ viæ and kidneys. In the former, the skin is evidently relaxed; and the histories of the dissections of diabetic patients, shew a similar laxity and softness of the kidneys. The perspiratory discharge in the former, when collected on a sponge, speedily acquires a sour odour; the urine in diabetes soon undergoes the same change. To these may be added, that in the *tabes sudatoria,* the urine is very scanty; while in diabetes the perspiratory discharge is, in a great measure, suppressed. The other symptoms are nearly the same; emaciation, debility, and hectic, equally mark both diseases. The analogy, then, which subsists between diabetes, and diseases usually ranked under the head of cachexiæ, would, as before-mentioned, strongly incline me to place it in that order."

If this analogy really exists, is it not a strong confirmation of Dr. Willan's remark, that he "never met with a confirmed case of diabetes, in which there was not some considerable disorder of the constitution, or a defect in some organ, essential to life."

Considerable disorder in the constitution cannot be questioned; but if there is always an organic affection, it increases the analogy remarked by Dr. Alley; as *tabes sudatorius* is generally a consequence of some other, and often incurable, disease.

